# Multi modal optical coherence tomography flowmetry of organ on chip devices

**DOI:** 10.1038/s41598-025-10089-9

**Published:** 2025-07-17

**Authors:** Devrim Tugberk, Konstantine Cheishvili, Peter Speets, William Quirós-Solano, Anish Ballal, Nikolas Gaio, Jeroen Kalkman

**Affiliations:** 1 Department of Imaging Physics, Delft University of Technology, Lorentzweg 1, Delft, The Netherlands; 2 BIOND Solutions B.V., Molengraaffsingel 10, Delft, The Netherlands; 3 Instituto Tecnológico de Costa Rica, Dulce Nombre 30109, Cartago, Costa Rica

**Keywords:** Lab-on-a-chip, Imaging and sensing

## Abstract

Organ-on-chip (OoC) systems are microfluidic devices for maintaining live tissue under physiologically relevant (flow) conditions. Imaging of structure and flow is important for the characterization of OoC device design and visualizing tissue/fluid interaction. Here, we present 3D tissue and flow imaging in an OoC device with multi-modal optical coherence tomography (OCT) using a combination of OCT structural imaging and flow imaging with Doppler OCT, number fluctuation dynamic light scattering OCT, and particle image velocimetry OCT. We demonstrate the feasibility of combined imaging of OoC tissue culture morphology and high flow velocities. We also measure low velocities in the OoC tissue well showing good agreement with computational fluid dynamics simulations. Our results open up the way for studying the effect of flow on living tissue in OoC devices.

## Introduction

Organ-on-chip (OoC) systems are microscopic-scale flow cells, i.e., microfluidic devices, that biomimic the in vivo microenvironment with embedded living tissues to personalize medicine^1^ and improve drug development trajectories^2^. These systems must be biocompatible, mechanically stable, and with flow commensurable to the physiological microenvironment.

OoC flow structures and geometry play a pivotal role in the design of microfluidic organ-on-chip (OoC) systems, especially for applications like cell culture, organ-on-chip models, and drug delivery systems. Careful optimization of flow cell dimensions and pump parameters is essential to achieve the desired flow rates, shear stresses, and fluid dynamics, which are critical to device performance, and to foster the optimal physiological conditions that cells experience in the human body^3^. Information on tissue morphology and structure is important in studying its development over time. For example, measuring tissue formation rate, morphology, and reaction to drugs are important parameters in OoC research. This information is often obtained through imaging.

In addition to structural information, flow information is important for two aspects of the OoC. First, characterizing the OoC flow speed distribution is needed to guide the design of the OoC device to make it operate under the right physiological conditions. For example, the OoC tissue well has to have a sufficiently high refresh rate to supply the tissue with enough nutrients. In addition, flow rates must be physiologically realistic so that drug distribution and cell interactions are similar to those in humans, something of key importance for drug testing and development. Second, for application to biological tissue, visualizing the flow-tissue interaction is crucial, for example, to quantify realistic dynamic flows or to measure the shear rate at the tissue-fluid interface and its impact on the development of epithelial cells.

Therefore, 3D monitoring of all components of the OoC device, such as cells, tissue, fluid flow, and microfluidic geometry, is of paramount importance to obtain information on OoC functioning. However, there are few technologies that combine the desired combination of 3D structural imaging (sample geometry and tissue morphology) with functional imaging (fluid flow and tissue perfusion).

Structural imaging has been performed with techniques such as brightfield microscopy, quantitative phase imaging, and fluorescence microscopy^4^ that mainly collect 2D structural information. Various 3D imaging techniques have been applied such as confocal fluorescence microscopy, selective plane illumination microscopy. However, fluorescence microscopy requires tissue labeling, whereas selective plane illumination microscopy requires sample access from all directions, something that is cumbersome for the planar geometry of OoC devices.

Flow imaging has been performed with brightfield microscopy combined with spatio-temporal image correlation spectroscopy and has shown good flow results in 2D^5^ and was implemented in 3D with selective plane illumination microscopy^6^. However, its implementation is rather cumbersome given the planar geometry of OoC devices. Quantitative flow imaging has been performed with particle image velocimetry (PIV), which is based on tracking fluorescent beads in a flow. Using imaging from multiple directions, the 3D velocity can be obtained^7^. However, PIV requires complicated equipment, a darkened measurement environment, and can only be performed in optically clear media. Moreover, it does not provide the corresponding structural information.

Optical coherence tomography (OCT) is very well suited for combined 3D structural and flow imaging of OoC devices. OCT is fast, label-free, and non-invasively images using a large working distance. OCT has been widely applied to image microfluidic systems, since it is ideally suited with an imaging depth of up to 2 mm in tissue and an axial resolution of a few micrometers. For OoC devices, OCT has been used for studying the lumen development in airways-on-chip^8^, thrombus formation in vessel-on-chip^9^, biofilm growth^10^, and bacterial colonization^11^. OCT also has been applied to image in vitro organoid development over time^12,13^. The high temporal resolution of OCT gives the ability of label-free high-contrast tissue imaging using speckle variance^9^. Moreover, OCT is a versatile imaging tool that can be used to measure tissue properties such as biological activity^12^, cellular reorganization^13^ and tissue mechanics^14^.

For quantitative OCT flow imaging, Doppler OCT is the most common approach due to its ease of implementation and ability to measure high flow speeds. However, Doppler OCT can only measure the axial flow component and the smallest measured flow speed is limited by the random signal from Brownian motion^15^. Particle image velocimetry OCT (PIV-OCT) has been used to measure both axial and transverse flow components in subsequent B-scans^16,17^. Although OCT-PIV can measure extremely small flows that are in arbitrary directions, it has lower spatial resolution, is computationally intensive, and requires cumbersome fine tuning of correlation windows to the target flow velocity components. Dynamic light scattering OCT (DLS-OCT) can measure both axial and lateral flow components^18^ but, similar to Doppler OCT, cannot measure low flow speeds as the Brownian motion causes random signal fluctuations. This problem has been addressed with number-fluctuation DLS-OCT^19^ where the signal fluctuations are caused by individual particles moving in and out of the focus, and the number-fluctuation part of the correlation function is independent of particle diffusion. Consequently, number-fluctuation DLS-OCT allows for the measurement of extremely low total flow speeds down to around ${50}\,{\upmu \text{m}}\,\text{s}^{-1}$. The different flow speeds, spatial resolution, and the requirement to assess the flow direction require the combined efforts of various OCT flow measurement techniques.

In this work, we demonstrate the versatility of OCT to measure, with multiple OCT operation modes the OoC geometry, tissue structure, and different flow speeds and directions. We do this by performing conventional OCT structural imaging and flow measurements with Doppler OCT, number-fluctuation DLS-OCT, and PIV-OCT. The flow speed and velocity in the OoC device well show good agreement with computational fluid-dynamic simulations.

## Methods

### OCT imaging

The experiments were done using a Thorlabs GANYMEDE II HR series spectral-domain OCT system^15^. The system bandwidth is centered at 900 nm and has an axial resolution of ${3} \, \upmu \text{m}$ in air. The OCT system is operated with an NA = 0.05 scan lens (LSM04-BB, Thorlabs). The beam waist $w_0= {6}\,\upmu \text{m}$ in air, defined as the $\text {e}^{-1}$ radius of the Gaussian field profile. The OCT system was used for structural imaging in B-scan mode. The OCT optical path length was converted to physical depth *z* using the refractive index of water $n_k=1.33$.

### OCT flow measurements

**Fig. 1:**
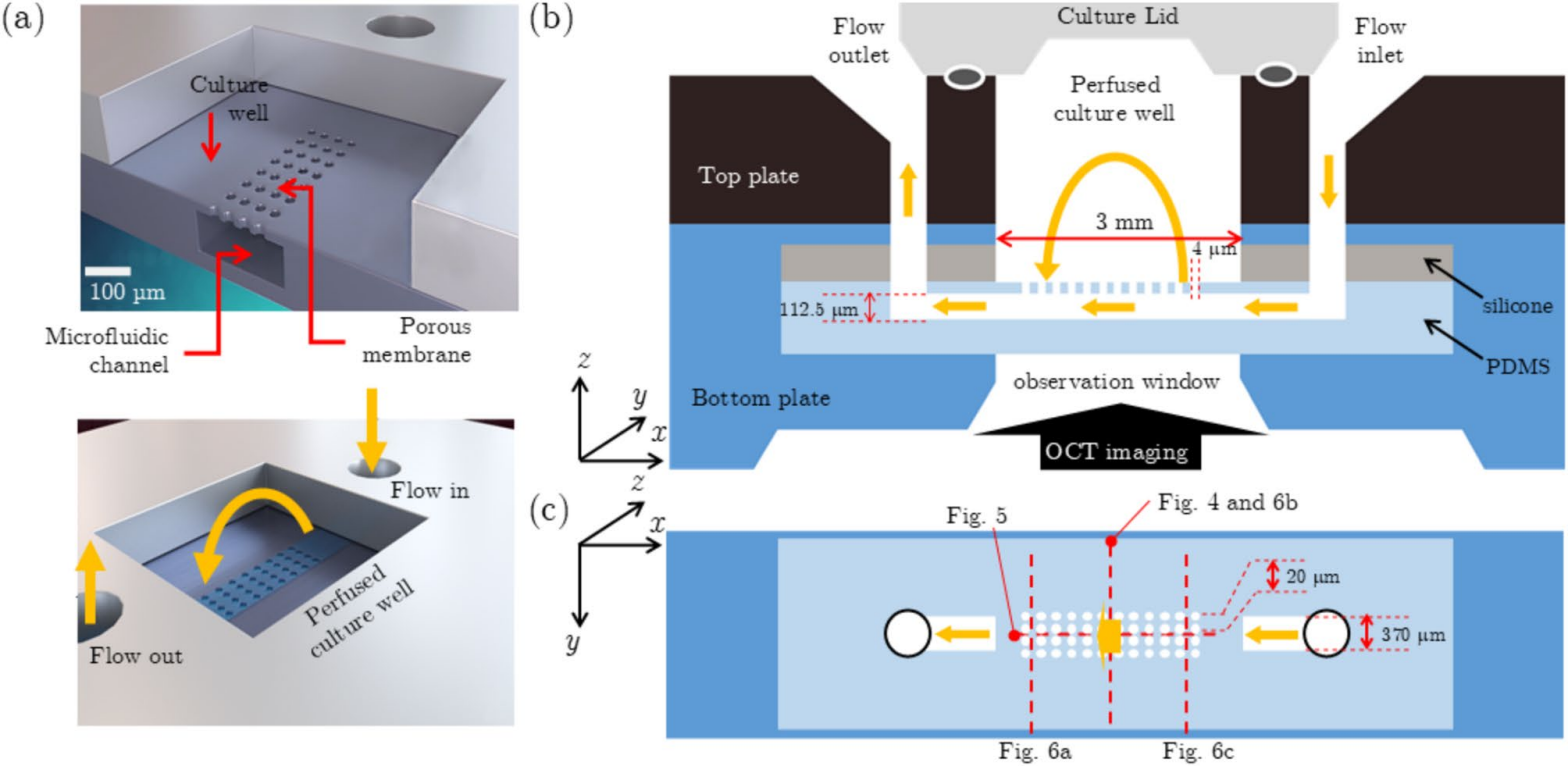
(**a**) Computer rendered images of the inCHIPit™-1C displaying the cross-section of the microfluidic channel, porous membrane, the static culture well, and the direction of flow from the inlet, through the microfluidic channel pores, creating a perfusion area, and back out from the outlet. (**b**) Cross-sectional view of the inCHIPit™-1C, the flow directions, dimensions, and OCT observation window. (**c**) Top view of the flow channel-well geometry with indicated the planes at which the various OCT measurements are performed.

The OCT system was used for imaging the OoC device through the observation window, see Fig. 1. The OCT system operated in Doppler mode for high flow velocities, such as in the OoC channels, and in number-fluctuation DLS-OCT and PIV-OCT mode for low flow velocities, such as in the OoC well.

For DLS-OCT and PIV-OCT the flow geometry was arranged so that the OCT beam was perpendicular to the microfluidic channel surface. Two-dimensional flow measurements were performed by laterally scanning the OCT beam (B-scan) along the width or length of the microfluidic channel. The scan length was 3.1 mm, covering 200 lateral pixels, while the number of axial pixels was 1024. A sequence of 2000 consecutive B-scans was obtained with a sampling time step of $\Delta t=40$ ms. The size of the pixels in the lateral and axial directions, $\Delta x$ and $\Delta z$, was 15.6 and 1.8 µm, respectively. DLS-OCT and PIV-OCT used 5.5 kHz A-scan rate. For these measurements, very dilute suspensions were used that were optimized to have a good balance between the number-fluctuation term magnitude in $g_2$ and the signal-to-noise ratio. The particle volume fraction used was between 0.005 and 0.01%. The pump discharge rate was ${40} \, \upmu \hbox {L min}^{-1}$.

#### DLS-OCT total velocity measurements

The in-plane total flow velocity was determined using number-fluctuation DLS-OCT^19,20^. The second-order normalized autocovariance function was computed from the OCT signal intensity time series at every lateral, *x*, and axial position, *z*. Due to a relatively large sampling time, diffusive decay could not be detected, and only the number-fluctuation term was obtained. The number-fluctuation intensity autocovariance function is

1 $$\begin{aligned} g_2(x, z, \tau ) = \frac{\left( \kappa (x,z)-1\right) \text {e}^{-\frac{{v_0(x,z)}^2\sin ^2{\theta (x,z)}\tau ^2}{w_z^2}}\text {e}^{-\frac{2{v_0(x,z)}^2\cos ^2{\theta (x,z)}\tau ^2}{{w_r(z)}^2}}}{\bigg (\kappa (x,z)-2+\Big (1+\frac{1}{\text {SNR}(x,z)}\Big )^2\bigg )\bigg (2^{3/2}N(x,z)+1\bigg )}\, , \end{aligned}$$

where $v_0(x,z)$ is the total velocity, $\text {SNR}(x,z)$ is the signal-to-noise ratio, *N*(*x*, *z*) is the average number of particles within the scattering volume, $w_r(z)$ is the local Gaussian beam waist, $w_z$ is the coherence function waist, $\theta (x,z)$ denotes the Doppler angle $(90^{\circ }-\theta$ is the angle between the velocity vector and the optical axis), and $\kappa (x,z)$ is the kurtosis of the noise-subtracted complex field distribution^20^. As seen in Eq. (1), a fit of the autocovariance function cannot distinguish between variations in velocity $v_0$ and variations in angle $\theta$. To eliminate the dependence of the autocorrelation function on the Doppler angle $\theta$, the width of the Gaussian spectral apodization window, $\sigma _k$, was varied to equalize the lateral and axial flow at each axial voxel, such that $\sqrt{2}w_z=w_r(z)$. Subsequently, the measured intensity autocorrelation functions were fitted using

2 $$\begin{aligned} g_2(x,z,\tau ) = A(x,z)\text {e}^{-{v_0(x,z)}^2\tau ^2\left( \frac{\sin ^2{\theta }}{{w_z(z)}^2}+\frac{2\cos ^2{\theta }}{{w_r(z)}^2}\right) }= A(x,z)\text {e}^{-\frac{2{v_0(x,z)}^2\tau ^2}{{w_r(z)}^2}}\,, \end{aligned}$$

where $A (x,z)$ is the amplitude factor for the intensity autocorrelation function incorporating all the pre-terms in Eq. (1). In this case, the fit parameters are $A (x,z)$ and $v_0(x,z)$.

**Fig. 2:**
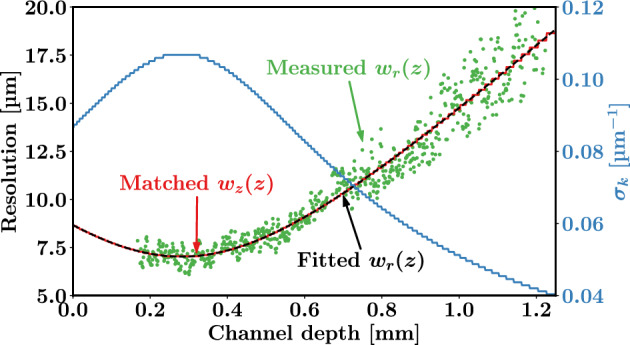
Measured and fitted Gaussian beam width as a function of depth, along with the matched coherence length and spectral apodization window.

For every axial voxel, a spectral apodization window width $\sigma _k(z)$ was implemented such that $\left( \sigma _k(z) n_k\right) ^{-1}=\sqrt{2}w_z(z)=w_r(z)$. The refractive index $n_k=1.33$ of water was used, as the aqueous particle suspension was very dilute. The effect of dispersion on the coherence length was neglected. The Gaussian beam shape, $w_r(z)$, was calibrated by scanning the beam over the stationary particle suspension, following the procedures described in Refs.^19,20^. The local beam waist, the matched coherence waist, and the spectral apodization window width are shown in Fig. 2. The beam shape $w_r(z)$ was fitted using $w_r(z)=w_0\sqrt{1+(z-z_0)^2/z^2_R}$. The measured beam shape from Fig. 2 is a good match with the Gaussian fit.

#### PIV-OCT directional velocity measurements

In-plane velocity vectors were determined by implementing PIV-OCT. Laterally and axially resolved OCT B-scan intensity images were divided into smaller windows, $I(\epsilon , \eta , t)$, each containing 4 lateral and 32 axial pixels covering a square area of ${62}\, \upmu \text{m}\,\times \,{44}\, \upmu \text{m}$. For each window, the time-dependent normalized unbiased 2D cross-covariance matrix was computed for every image pair using

3 $$\begin{aligned} \rho (m,n,t) = \frac{\left\langle \left( I(\epsilon , \eta , t)-\overline{I}_{\epsilon , \eta }(t)\right) \left( I(\epsilon +m, \eta +n, t+N\Delta t)-\overline{I}_{\epsilon , \eta }(t+N\Delta t)\right) \right\rangle _{\epsilon ,\eta }}{\sigma _{\epsilon ,\eta }(t)\sigma _{\epsilon ,\eta }(t+N\Delta t)\left( 4-|m|\right) \left( 32-\left| n\right| \right) }\,, \end{aligned}$$

where $\epsilon ,\eta$ are lateral and axial pixel numbers within the window reference frame, $\sigma _{\epsilon ,\eta }$ is the standard deviation over a window, *m* and *n* represent the relative pixel shifts in the corresponding directions, and *N* is the number of B-scans by which two frames are temporally separated. The obtained correlation coefficients were temporally averaged, resulting in the mean 2D cross-correlation coefficient $\overline{\rho }_t(m,n)$.

For each window, the lateral velocity $v_x$ was computed by finding the lateral index of the maximum of $\overline{\rho }_t(m,n)$, denoted as $m_\text {max}$, while the axial velocity was determined by the axial index of the maximum, denoted as $n_\text {max}$. The lateral and axial velocities were then determined using

4 $$\begin{aligned} v_x = \frac{m_\text {max}\Delta x}{N\Delta t} \qquad \text {and} \qquad v_z = \frac{n_\text {max}\Delta z}{n_kN\Delta t}\,. \end{aligned}$$

In our analysis, we used $N=5$ to calculate $v_z$ and $N=20$ to calculate $v_x$, except at the center of the channel, where we used $N=15$ to calculate both velocity components. The minimum sampling time between two images is 202 ms when $N=5$. This is significantly larger than the time required for the acquisition of the intensity image of one window, which is approximately 0.7 ms. Therefore, particle motion within this time window was neglected.

### Doppler OCT measurements

Doppler OCT measurements were performed on the main channel of the device channels where the flow was high. In two separate OoC devices, human umbilical vein endothelial cells (HUVECs) were cultured in the microfluidic channel and arising retinal pigment epithelial (ARPE19) were cultured in the static culture well, respectively. The cells grew completely through the pores into the microfluidic channel thereby blocking the openings/access to the culture well, making these devices resemble the behavior of a microfluidic channel with blocked/without pores. Upon reaching a stable and mature adhesion to PDMS, all cell cultures were fixed with paraformaldehyde. Due to biosafety regulations and compliance to OoC device handling with GMO (genetically modified organisms) cultivated cell lines, the measurements were performed on dead tissue.

We measured flow with Doppler-OCT in the main flow channel using Intralipid diluted to 0.06% volume fraction particles as tracer particles in two different OoCs. The pump discharge rate was ${25} \, {\upmu \hbox {L min}}^{-1}$. The Doppler angle determined from the alignment in a set of B-scans was $10.5^{\circ }$. Subsequently, it was compensated for refraction of the OoC observation window. Doppler measurements were performed in B-scan mode using 20000 A-scans at 36 kHz scan rate over the ${400}\,{\upmu \text{m}}$ channel width and averaged 20 times. Doppler-OCT flow measurements were implemented using the phase-resolved method using B-scans as described by Cheishvili et al.^15^.

### Organ-on-chip samples

Flow measurements were performed on inCHIPit™ OoC devices (inCHIPit™–1C, BIOND Solutions B.V., Delft, the Netherlands). The inCHIPit™ OoC device consists of a single poly(dimethylsiloxane) (PDMS) microfluidic channel, with a porous membrane on the ceiling of the channel that leads to a static culture well, see Fig. 1(a). The flow going in and out of the system, via the inlet and outlet respectively, is the driving force of fluid movement inside the system and the porous membrane geometry creates an active perfusion flow inside the culture well. The cross section and top view of the device are shown in Fig. 1(b) and (c), respectively.

PDMS is a soft polymer used in the inCHIPit™ device due to its bio-compatibility, gas permeability, optical transparency, chemical inertness, elasticity, and low costs^21^. The hydrophobic surface of PDMS originates from the presence of the organic methyl groups, causes clogging of the membrane pores. Therefore, we increased the hydrophilicity of PDMS via plasma surface modification^22^. We treated the surface with an oxygen plasma (0.22 mBar) for 3 minutes (Atto, Diener, Germany). Exposure to the oxygen plasma replaces the methyl groups on the PDMS with hydroxyl groups to create polar silanol groups, making the surface hydrophilic. This can decrease the water contact angle of PDMS by 30° or more, depending on the treatment time, significantly improving cell adhesion to the surface and increasing the ease with which fluid, and its constituents, passes through the porous PDMS membrane. The increased PDMS hydrophilicity in combination with appropriate cell-adhesive coating and the aforementioned standalone benefits of PDMS create a microenvironment that allows cell culturing. The hydrophilic effects induced via plasma treatment are temporary and wear off depending on factors such as the PDMS chemistry and OoC storage method. The hydrophilic hydroxyl groups are highly reactive and can react with molecules found in air and revert back to a hydrophobic state in 1-2 days for our PDMS.

### Computational fluid dynamics simulations

**Fig. 3:**
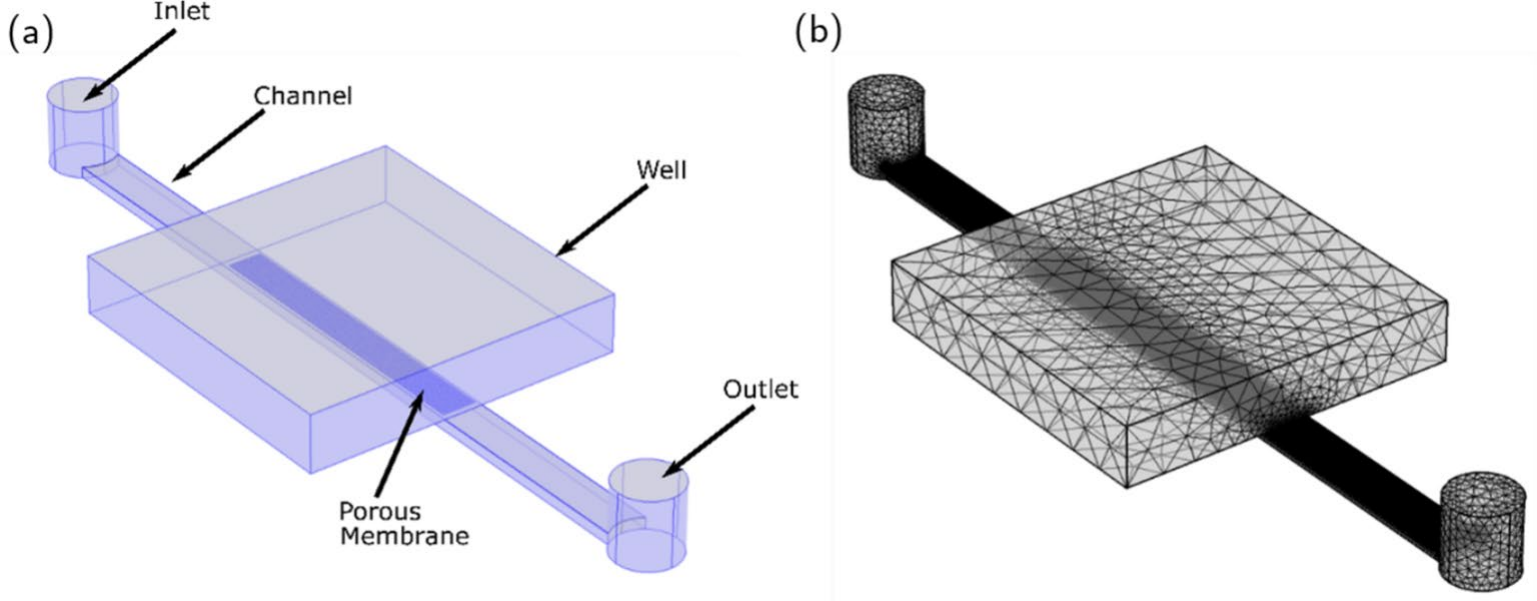
(**a**) Geometry of the inCHIPit™ in COMSOL Multiphysics and the corresponding boundary conditions set for the computation of the velocity field under steady-state conditions. (**b**) Meshed geometry of the inCHIPit™ in COMSOL Multiphysics.

Numerical computational fluid dynamics (CFD) simulations were performed using finite element method software COMSOL Multiphysics®v5.6 to model the flow behavior of the inCHIPit™ OoC device from BIOND Solutions B.V. The developed model computationally solved the Navier-Stokes equation neglecting the inertial term. This is valid since the Stokes flow problem is considered to be under steady-state pressure-driven conditions and is expected to remain under steady-state for the envisioned applications of the device. The OoC device geometry comprises five main parts: inlet, outlet, microchannel, porous membrane, and well. The dimensions of these parts are set according to the measured lengths of the inCHIPit™ device and are shown in Table 1. Figure 3(a) shows an example of the simulated flow geometry. The corresponding boundary conditions along with the material properties were set. Water was the fluid of interest with a viscosity value 10^-3^ Pa$\cdot$s at 20 $^{\circ }$C. A boundary condition of laminar flow was set at the inlet for the different flow rates of interest. At the outlet, an open boundary with null normal stress was used. The walls of the well, channels and porous membrane were set to a no-slip condition for the fluid velocity.

**Table 1:** Parts and dimensions of the computationally modeled inCHIPit™ device.

Part	Feature	Dimension [${\upmu \text{m}}$]
Inlet and outlet	Diameter	600
	Height	630
Microchannel	Length	7200
	Width	370
	Height	112.5
Porous membrane	Thickness	6
	Pore diameter	4
	Pore spacing	20
Well	Length	3000
	Width	3000
	Height	525

The geometry was meshed with 6012570 elements with an average element quality of 0.6854 for different element types (tetrahedral, triangular). An image of the meshed geometry is shown in Fig. 3(b). The computation was carried out with a server with two cores Intel(R) Xeon(R) CPU E-54667 v3 @ 2.00GHz and 32 GB RAM memory, for an average computation time of 3 hours for most of the simulations. All components of the flow speed were calculated and used for comparison to the measurements.

## Results

### Flow in a single channel OoC system

To show the versatility of combined OCT morphology and flow measurements, we first studied cell morphology and flow inside the OoC device.

**Fig. 4:**
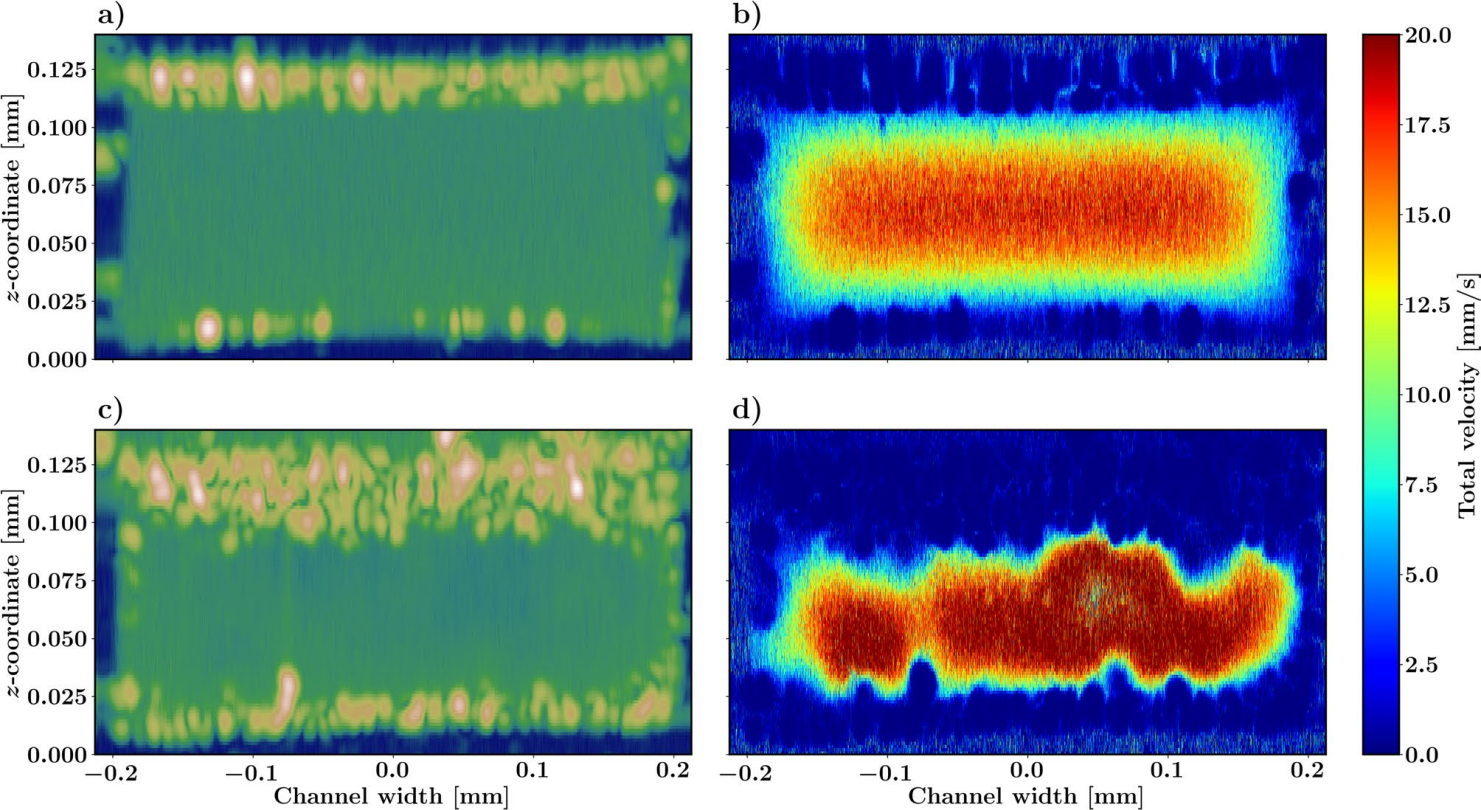
Cross-sectional B-scan intensity in dB scale perpendicular through the flow channel for the channel (**a**) with no pores and (**c**) with the presence of epithelial cells protruding from the culture well into the channel through the porous membrane. (**b**, **d**) Corresponding Doppler OCT flow measurements of the lumen.

Figures 4(a) and (b) show the OCT structural and flow speed images of the open channel. This section of the open channel has no pores nor cells and shows some minor elastic channel deformation. The flow is laminar and the flow speed is calculated using the Doppler angle of the OoC flow channel. Figures 4(c) and (d) show the structural and flow OCT image of a section of the channel constricted by the growing tissue. In the constrained channel, epithelial cells grow inside the culture well and clog the porous membrane. The cells essentially make the OoC act as a single channel without pores. Even though the contrast between the original channel and the cell monolayer is low, the flow channel can still be observed in the structural image. The flow image in Fig. 4(d) clearly shows the channel constriction as the channel lumen becomes smaller and the flow speed increases (measurement at constant discharge rate). Note that in the case of lumen restrictions, the flow does not necessarily need to be in the direction of the flow channel, and the absolute flow speed may be slightly off.

Although the Doppler OCT method is easy to implement and can measure the flow in the channels without much problems, measuring the flow speed in the OoC well with Doppler OCT was close to impossible under realistic flow conditions. Hence, we implemented number-fluctuation DLS-OCT and PIV-OCT to measure the flow also in these spaces with the same OCT system.

### Flow in the OoC well

**Fig. 5:**
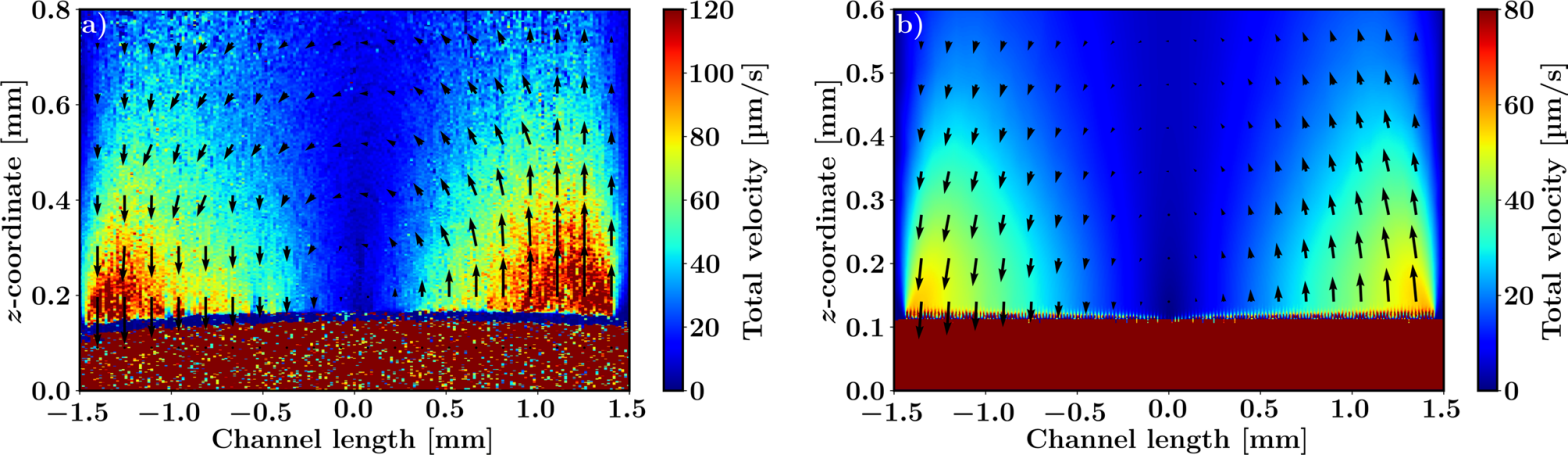
(**a**) Total velocity distribution along the channel length derived from number fluctuation DLS-OCT with superimposed in-plane velocity vectors measured using PIV-OCT. (**b**) Corresponding CFD simulations.

**Fig. 6:**
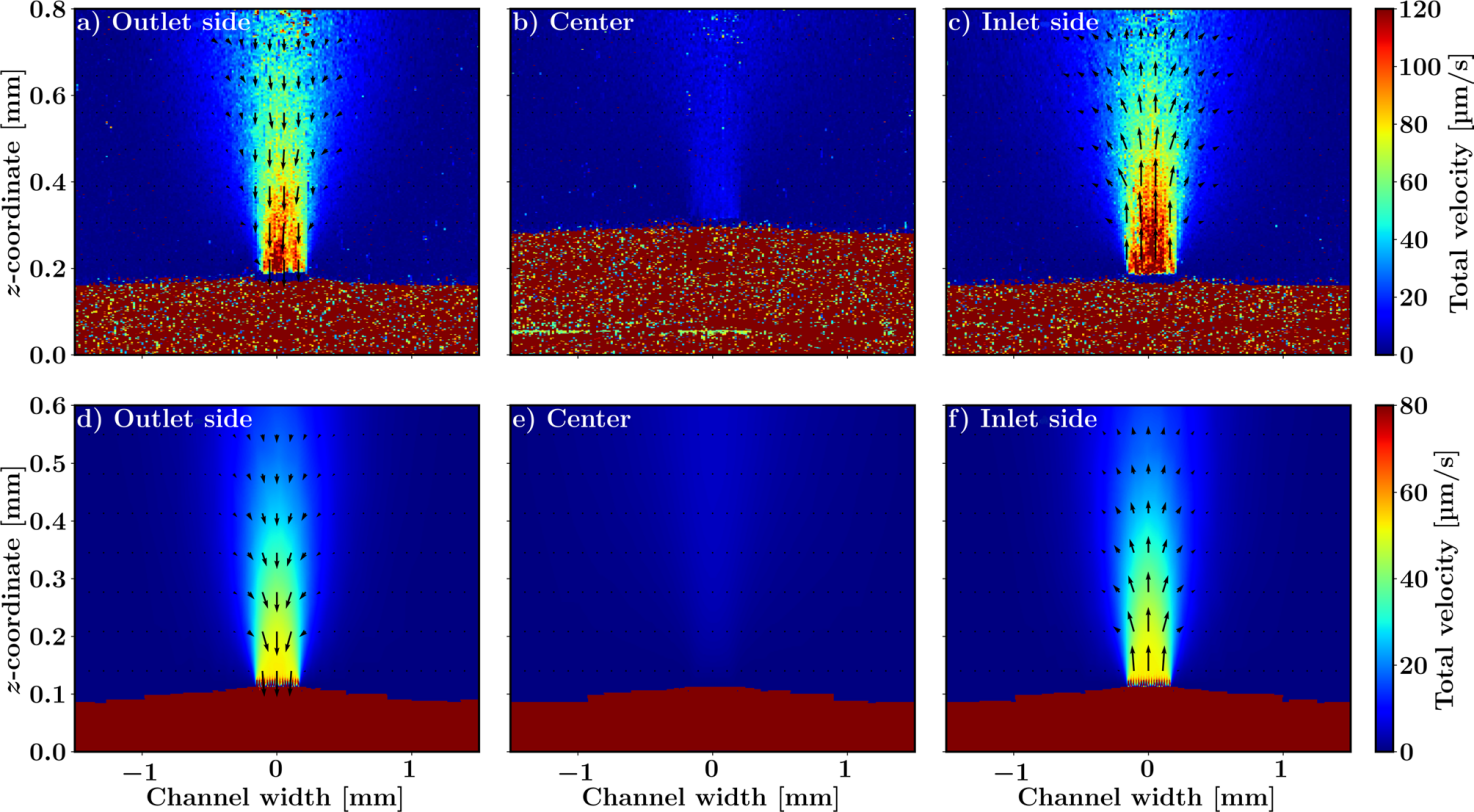
(**a**–**c**) Total velocity distribution along the channel width derived from number fluctuations, accompanied by in-plane velocity vectors measured using particle tracking. (**d**–**f**) Corresponding CFD simulations.

Figures 5 and 6 show a comparison of the CFD simulations to combined number-fluctuation DLS-OCT and PIV-OCT measurements inside the OoC culture well. The flow profile along the symmetry plane along the microchannel from the inlet to the outlet is shown in Fig. 5(a) for the OCT measurements and in Fig. 5(b) for the CFD simulations. The presented measurements are only sensitive to the low flow speeds in the well; the higher flow speeds in the microchannel can be quantified separately with Doppler OCT (similar to the data in Fig. 4). The flow enters the channel from the right where most of the flow enters the well through the porous membrane. In the well, the flow spreads out and the flow speed decreases. Near the outlet the flow speed again increases as the fluid goes out from the well into the channel. Overall, there is good qualitative agreement between the simulations and the measurements showing similar flow directions as well as flow distributions. There is less absolute quantitative agreement, with the simulated flow speeds being approximately 50% lower than the measured flow speeds.

The flow profile perpendicular to the line from the inlet to the outlet in Fig. 6 shows the flow entering the well at the inlet and exiting the well from the outlet. In the middle of the well, the *z*-component of the flow is almost completely absent, which is what one would expect for this plane. Again, there is good qualitative agreement between the simulations and the measurements showing similar flow directions as well as flow distributions, but less absolute quantitative agreement, with the simulated flow speeds being approximately 50% lower than the measured flow speeds. Note that the measurements in the center clearly show the bulging out of the porous membrane from the channel into the well caused by the fluid pressure acting on the flexible PDMS porous membrane.

## Discussion

We demonstrated structural and functional measurements in an OoC system using multiple OCT imaging modes such as OCT, Doppler-OCT, PIV-OCT and DLS-OCT flow.

Our demonstration of in vitro OoC Doppler-OCT flow measurements, as shown in Fig. 4, opens up a way to study the effect of relatively high flows on in vivo tissue growth and development. The flow stimulates the cells residing on the cell-adhesive coated substrate with flow-induced shear stress whereas the pores provide a 3D microenvironment where the cells can sense the architecture and attach accordingly. In addition to flow being an excellent flow/tissue contrast mechanism, the greatest value for such OCT measurements is in the quantification of blood flow and shear rates. For example, OCT flow measurements in the (micro) vasculature of vessel-on-chip or tumor-on-chip may be used to measure the shear rate in vivo. Shear rate is a biologically relevant stimulus for vessel modification sensed by the epithelial cells lining the vessel wall, which is important in the study of atherosclerosis, effects of high blood pressure, and vascular remodeling.

We successfully demonstrated the challenging task of measuring the low flow speed and velocities present in the OoC well. Comparison with CFD calculations, as shown in Figs. 5(a) and 6(a–c), gave good qualitative agreement, however, quantitatively, the measured velocities are approximately 1.5 times greater than those in the simulations. We attribute this disagreement to a flow geometry that is not identical to the rectangular designed shape, as we observed that due to the pump pressure the elastic PDMS membrane deformed, as can be seen in the elevation in the middle of the flow channel in Fig. 6(b) and (e). The bulging of the channel is mainly due to the lack of mechanical support in the center of the channel and the low pressure inside the culture well (relative to the microchannel) due to the partially sealing lid that isolates the culture well from the environment. The lid allows air to escape from the culture well, which creates a small air leak in the system that causes the channel to bulge into the culture well due to the relatively low pressure and consequently this leads to more fluid traveling through the culture well and hence a higher velocity. Another consequence of the bulging channel is the variability in the pore sizes of the porous membrane along the channel. These variations are difficult to model, emphasizing the need for in situ flow measurements for flow performance calibration of OoC devices. Alternatively, the stiffness of the PDMS can be increased to avoid changes in flow geometry under pressure^23^.

In Fig. 5(a) and 6(a–c), we use both number-fluctuation DLS-OCT, to measure the total flow speed, and PIV-OCT for directional velocities, from the same data. The magnitude of the total velocity obtained with PIV-OCT matches well to the values obtained with number-fluctuation measurements. Although PIV-OCT offers the advantage of capturing in-plane 2D velocity vectors, it has some drawbacks over DLS-OCT. First, the spatial resolution of PIV-OCT flow imaging is significantly reduced due to the required spatial windowing which is especially prominent when the flow is highly confined, such as in the well inflow and outflow regions. Second, PIV-OCT requires careful tuning of the temporal separation between windows ($N\Delta t$), the temporal window size, and the lateral window size to be able to measure a particular flow speed and direction. Third, the PIV-OCT 2D cross-correlation analysis causes a large computational overhead. Number-fluctuation DLS-OCT is a more robust method, requires no parameter optimization, and is readily applicable to the original resolution of the data obtained. However, it is only sensitive to the total velocity and cannot capture individual velocity components. The combination of PIV-OCT and number fluctuation DLS-OCT can be used to get good low resolution quantitative assessment of the flow direction with number fluctuation DLS-OCT supplementing this with high resolution total flow speed data.

Stable and consistent flow measurements of an OoC with pores turned out to be challenging due to particles clogging the pores of the system. The flow through the system was rapidly fluctuating due to the clogging of pores and the consequential build-up of pressure that resulted in some pores becoming un-clogged and clogged again. After various experimental trials, it was deduced that the highly hydrophobic nature of the PDMS caused clogging. We could visibly observe that the highly hydrophobic surface of PDMS essentially created such a high particle repelling force that the $4\,{\upmu \text{m}}$ pores of the system were not accessible for the particles from the suspension. Even particles of 200 nm in diameter would not pass through the pores, either creating clogs or simply rolling over the pores. The clogging of the pores created unstable flow environments within the OoC and made reproducible OCT measurements impossible due to the sporadic nature of the clogging. This problem was overcome with surface plasma treatment of the entire OoC, which is a standard treatment procedure for OoCs to improve cell adhesion on PDMS. After plasma treatment the flow was stable for many hours. Experiments could be repeated in a time frame of up to a few days, after that plasma-treatment effects wear off over time. Fortunately, many other techniques have been developed to address this issue in the future^24^.

In conclusion, we demonstrated versatile multi-mode OCT structural and flow imaging in an OoC device. Doppler-OCT, PIV-OCT, and number-fluctuation DLS-OCT supplement each other and are applicable to image the flow and velocity in different speed regimes that are in different parts of the OoC device.

## Data Availability

The datasets generated and/or analyzed during the current study are available in the Zenodo repository https://doi.org/10.5281/zenodo.14293638. For questions regarding the dataset and analysis methods please contact the corresponding author at j.kalkman@tudelft.nl.

## References

[1] van den Berg, A., Mummery, C. L., Passier, R. & van der Meer, A. D. Personalised organs-on-chips: Functional testing for precision medicine. *Lab Chip* **19**, 198 (2019). 30506070 10.1039/c8lc00827b PMC6336148

[2] Esch, E. W., Bahinski, A. & Huh, D. Organs-on-chips at the frontiers of drug discovery. *Nat. Rev. Drug Discov.* **14**, 248–260 (2015). 25792263 10.1038/nrd4539 PMC4826389

[3] Pisapia, F., Balachandran, W. & Rasekh, M. Organ-on-a-chip: Design and simulation of various microfluidic channel geometries for the influence of fluid dynamic parameters. *Appl. Sci.* **12** (2022).

[4] Buchanan, B. C. & Yoon, J. Y. Microscopic imaging methods for organ-on-a-chip platforms. *Micromachines* **13**, 328 (2022). 35208453 10.3390/mi13020328 PMC8879989

[5] Travagliati, M., Girardo, S., Pisignano, D., Beltram, F. & Cecchini, M. Easy monitoring of velocity fields in microfluidic devices using spatiotemporal image correlation spectroscopy. *Anal. Chem.* **85**, 8080–8084 (2013). 23919917 10.1021/ac4019796

[6] Ceffa, N. G. et al. Spatiotemporal image correlation analysis for 3D flow field mapping in microfluidic devices. *Anal. Chem.* **90**, 2277–2284 (2018). 29266924 10.1021/acs.analchem.7b04641

[7] Elsinga, G. E., Scarano, F., Wieneke, B. & van Oudheusden, B. W. Tomographic particle image velocimetry. *Exp. Fluids* **41**, 933 (2006).

[8] Liu, Z. et al. Co-cultured microfluidic model of the airway optimized for microscopy and micro-optical coherence tomography imaging. *Biomed. Opt. Express* **10**, 5414 (2019). 31646055 10.1364/BOE.10.005414 PMC6788592

[9] Cuartas-Vélez, C., Middelkamp, H. H. T., van der Meer, A. D., van den Berg, A. & Bosschaart, N. Tracking the dynamics of thrombus formation in a blood vessel-on-chip with visible-light optical coherence tomography. *Biomed. Opt. Express* **14**, 5642 (2023). 38021142 10.1364/BOE.500434 PMC10659801

[10] Weiss, N., El Tayeb El Obied, K., Kalkman, J., Lammertink, R. & van Leeuwen, T. Measurement of biofilm growth and local hydrodynamics using optical coherence tomography. *Biomed. Opt. Express* **7**, 3508 (2016). 10.1364/BOE.7.003508 PMC5030028 27699116

[11] Yuan, L. et al. Visualization of bacterial colonization and cellular layers in a gut-on-a-chip system using optical coherence tomography. *Microsc. Microanal.* **26**, 1211 (2020). 33107427 10.1017/S143192762002454X

[12] Ming, Y. et al. Longitudinal morphological and functional characterization of human heart organoids using optical coherence tomography. *Biosens. Bioelectron.* **207**, 114136 (2022). 35325716 10.1016/j.bios.2022.114136 PMC9713770

[13] Monfort, T. et al. Dynamic full-field optical coherence tomography module adapted to commercial microscopes allows longitudinal in vitro cell culture study. *Commun. Biol.* **6**, 992 (2023). 37770552 10.1038/s42003-023-05378-w PMC10539404

[14] Mason, J. H. et al. Debiased ambient vibrations optical coherence elastography to profile cell, organoid and tissue mechanical properties. *Commun. Biol.* **6**, 543 (2023). 37202417 10.1038/s42003-023-04788-0 PMC10195840

[15] Cheishvili, K. & Kalkman, J. Scanning dynamic light scattering optical coherence tomography for measurement of high omnidirectional flow velocities. *Opt. Express* **30**, 23382 (2022). 36225019 10.1364/OE.456139

[16] Jonas, S., Bhattacharya, D., Khokha, M. K. & Choma, M. A. Microfluidic characterization of cilia-driven fluid flow using optical coherence tomography-based particle tracking velocimetry. *Biomed. Opt. Express* **2**, 2022 (2011). 21750777 10.1364/BOE.2.002022 PMC3130586

[17] Buchsbaum, A. et al. Optical coherence tomography based particle image velocimetry (OCT-PIV) of polymer flows. *Opt. Lasers Eng.* **69**, 40 (2015).

[18] Weiss, N., van Leeuwen, T. G. & Kalkman, J. Localized measurement of longitudinal and transverse flow velocities in colloidal suspensions using optical coherence tomography. *Phys. Rev. E* **88**, 042312 (2013). 10.1103/PhysRevE.88.042312 24229177

[19] Cheishvili, K. & Kalkman, J. Sub-diffusion flow velocimetry with number fluctuation optical coherence tomography. *Opt. Express* **3**, 3755 (2023). 10.1364/OE.474279 36785361

[20] Cheishvili, K., Rieger, B. & Kalkman, J. Precision and bias in dynamic light scattering optical coherence tomography measurements of diffusion and flow. *Biomed. Opt. Express* **15**, 1288 (2024). 10.1364/OE.521702 38859117

[21] Kutluk, H., Bastounis, E. E. & Constantinou, I. Integration of extracellular matrices into organ-on-chip systems. *Adv. Healthcare Mater.* **12**, 2203256 (2023). 10.1002/adhm.202203256 PMC11468608 37018430

[22] Zhou, J., Ellis, A. V. & Voelcker, N. H. Recent developments in PDMS surface modification for microfluidic devices. *Electrophoresis* **31**, 2–16 (2010). 20039289 10.1002/elps.200900475

[23] Seghir, R. & Arscott, S. Extended PDMS stiffness range for flexible systems. *Sens. Actuators A* **230**, 33–39 (2015).

[24] Neves, L. B. et al. A review of methods to modify the PDMS surface wettability and their applications. *Micromachines* **15** (2024). 10.3390/mi15060670 PMC11205751 38930640

